# New Insights into Mechanisms and Functions of Chemokine (C-X-C Motif) Receptor 4 Heteromerization in Vascular Smooth Muscle

**DOI:** 10.3390/ijms17060971

**Published:** 2016-06-20

**Authors:** Ann E. Evans, Abhishek Tripathi, Heather M. LaPorte, Lioubov I. Brueggemann, Abhay Kumar Singh, Lauren J. Albee, Kenneth L. Byron, Nadya I. Tarasova, Brian F. Volkman, Thomas Yoonsang Cho, Vadim Gaponenko, Matthias Majetschak

**Affiliations:** 1 Burn and Shock Trauma Research Institute, Department of Surgery, Loyola University Chicago Stritch School of Medicine, 2160 S. First Avenue, Maywood, IL 60153, USA; anevans@lumc.edu (A.E.E.); omnamahshiva80@gmail.com (A.T.); hlaporte@luc.edu (H.M.L.); lalbee@luc.edu (L.J.A.); 2 Department of Molecular Pharmacology and Therapeutics, Loyola University Chicago Stritch School of Medicine, 2160 S. First Avenue, Maywood, IL 60153, USA; lbruegg@luc.edu (L.I.B.); kbyron@luc.edu (K.L.B.); 3 Edward A. Doisy Department of Biochemistry and Molecular Biology, Saint Louis University School of Medicine, 1100 South Grand Blvd., St. Louis, MO 63104, USA; singh@slu.edu (A.K.S.); ycho9@slu.edu (T.Y.C.); 4 Cancer and Inflammation Program, National Cancer Institute, PO Box B, Frederick, MD 21702-1201, USA; tarasovn@mail.nih.gov; 5 Department of Biochemistry, Medical College of Wisconsin, 8701 Watertown Plank Road, Milwaukee, WI 53226, USA; bvolkman@gmail.com; 6 Department of Biochemistry and Molecular Genetics, University of Illinois at Chicago, 900 S Ashland, Chicago, IL 60607, USA; vadimg@uic.edu

**Keywords:** atypical chemokine receptor 3, (C-X-C motif) receptor 7, adrenergic receptor, vascular function, blood pressure, G protein-coupled receptor, receptor heteromer, structural modeling

## Abstract

Recent evidence suggests that C-X-C chemokine receptor type 4 (CXCR4) heteromerizes with α_1A/B_-adrenoceptors (AR) and atypical chemokine receptor 3 (ACKR3) and that CXCR4:α_1A/B_-AR heteromers are important for α_1_-AR function in vascular smooth muscle cells (VSMC). Structural determinants for CXCR4 heteromerization and functional consequences of CXCR4:α_1A/B_-AR heteromerization in intact arteries, however, remain unknown. Utilizing proximity ligation assays (PLA) to visualize receptor interactions in VSMC, we show that peptide analogs of transmembrane-domain (TM) 2 and TM4 of CXCR4 selectively reduce PLA signals for CXCR4:α_1A_-AR and CXCR4:ACKR3 interactions, respectively. While both peptides inhibit CXCL12-induced chemotaxis, only the TM2 peptide inhibits phenylephrine-induced Ca^2+^-fluxes, contraction of VSMC and reduces efficacy of phenylephrine to constrict isolated arteries. In a Cre-loxP mouse model to delete CXCR4 in VSMC, we observed 60% knockdown of CXCR4. PLA signals for CXCR4:α_1A/B_-AR and CXCR4:ACKR3 interactions in VSMC, however, remained constant. Our observations point towards TM2/4 of CXCR4 as possible contact sites for heteromerization and suggest that TM-derived peptide analogs permit selective targeting of CXCR4 heteromers. A molecular dynamics simulation of a receptor complex in which the CXCR4 homodimer interacts with α_1A_-AR via TM2 and with ACKR3 via TM4 is presented. Our findings further imply that CXCR4:α_1A_-AR heteromers are important for intrinsic α_1_-AR function in intact arteries and provide initial and unexpected insights into the regulation of CXCR4 heteromerization in VSMC.

## 1. Introduction

G protein-coupled receptors (GPCR) comprise the largest group of cell surface receptors and mediate a multitude of important cell functions. It is well established that the physiological range of GPCR-mediated signaling events and functions is amplified through cross-talk between GPCRs [1,2,3,4,5,6]. One molecular mechanism underlying GPCR cross-talk is receptor hetero-oligomerization, which is thought to be of importance for many aspects of GPCR function [7,8,9,10,11]. The structural determinants of GPCR heteromerization, however, are largely unknown, and the biological relevance of heteromeric receptor complexes is still under debate.

Multiple lines of evidence suggest that chemokine receptors and adrenergic receptors (AR) may form heteromeric receptor complexes with receptors of the same family and that chemokine receptors may also form heteromeric complexes with adrenergic receptors [10,12,13,14,15,16,17,18,19,20,21,22,23,24,25,26,27,28,29]. More recently, we provided evidence that C-X-C chemokine receptor 4 (CXCR4) forms heteromeric complexes with atypical chemokine receptor 3 (ACKR3) and α_1A/B_-adrenergic receptors (AR) on the cell surface of vascular smooth muscle cells (VSMC). As interference with CXCR4:α_1A/B_-AR heteromerization inhibited α_1_-AR mediated responses of VSMC, these data suggested that CXCR4:α_1A/B_-AR heteromerization is important for normal α_1_-AR function. Furthermore, we have previously reported that a peptide analog of transmembrane helix (TM) 2 of CXCR4 disturbs heteromeric complex formation between CXCR4 and α_1A/B_-AR, but does not influence CXCR4:ACKR3 heteromerization [30]. Whether it is possible to interfere with CXCR4:ACKR3 heteromerization without affecting CXCR4:α_1A/B_-AR heteromers, however, is unknown and the effects of CXCR4:α_1A/B_-AR heteromerization in VSMC on the function of intact arteries or on blood pressure regulation remain to be determined.

Here we show that CXCR4:α_1A_-AR and CXCR4:ACKR3 can be selectively targeted utilizing peptide analogs of transmembrane domain (TM) 2 and 4 of CXCR4, respectively, suggesting TM2 and TM4 of CXCR4 as possible contact sites in heteromeric receptor complexes. Accordingly, we modeled a hetero-hexameric receptor complex in which the CXCR4 homodimer interacts with α_1A_-AR via TM2 and with ACKR3 via TM4. Furthermore, we provide evidence that CXCR4:α_1A/B_-AR heteromers regulate intrinsic α_1_-AR function in intact arteries and new insights into the regulation of CXCR4 heteromerization in vascular smooth muscle from observations in a Cre-loxP mouse model.

## 2. Results

### 2.1. Transmembrane Domain-Derived Peptide Analogs of CXCR4 Selectively Interfere with Receptor Proximity and Function

**Figure 1:**
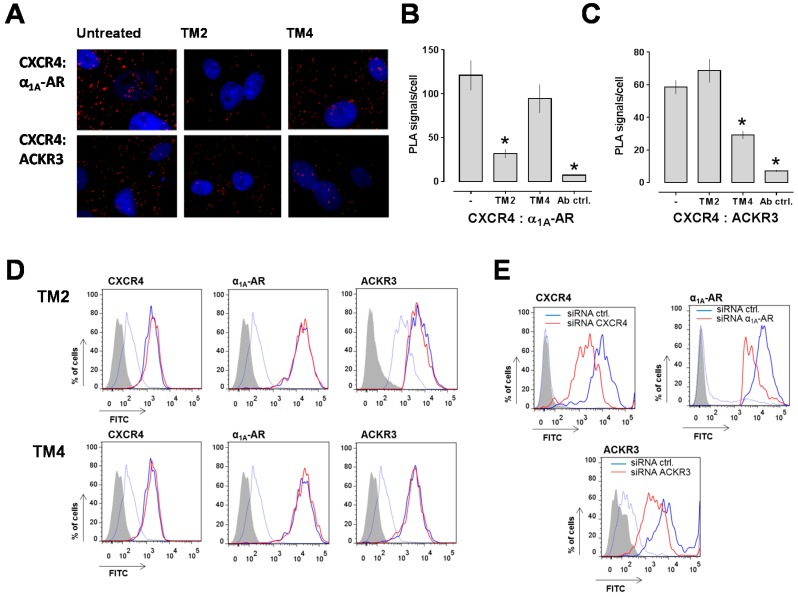
(**A**) Interactions between CXCR4 and α_1A_-AR (**top**) and between CXCR4 and ACKR3 (**bottom**) were visualized by PLA. Images show merged PLA/4′,6-diamidino-2-phenylindole dihydrochloride (DAPI) signals and are representative of 3 independent experiments. hVSMC were incubated with or without TM peptides (100 μM, 15 min at room temperature) prior to PLA; (**B**) Quantification of the number of PLA signals per cell for CXCR4:α_1A_-AR interactions as in A. Ab ctrl.: Antibody control; the primary antibodies were omitted. *n* = 3 (10 randomly selected non-overlapping vision fields were analyzed per experiment). *: *p* < 0.05 *vs.* untreated cells; (**C**) Quantification of the number of PLA signals per cell for CXCR4:ACKR3 interactions as in A. Ab ctrl.: Antibody control; the primary antibodies were omitted. *n* = 3 (10 randomly selected non-overlapping vision fields were analyzed per experiment). *: *p* < 0.05 *vs.* untreated cells; (**D**) FACS analyses of receptor cell surface expression levels in hVSMC after incubation with the TM peptides (100 μM, 15 min at room temperature). Grey: Unstained cells; Thin blue line: IgG control; Thick blue line: Cells incubated without TM peptides; Thick red line: Cells incubated with TM peptides; FITC: Fluorescein isothiocyanate; (**E**) FACS analyses of receptor cell surface expression after incubation hVSMC with non-targeting siRNA (thick blue line) or siRNA specific for each receptor (thick red line). Grey: Unstained cells; Thin blue line: IgG control.

To test whether TM peptide analogs may permit selective targeting of CXCR4 heteromers, we sought to evaluate the effects of TM peptide analogs of CXCR4 in PLA with hVSMC. The TM1 peptide analog, however, had solubility below 0.1 μM and the TM3 peptide analog was insufficiently hydrophobic to insert into a membrane bilayer, which precluded testing in VSMC assays [31]. Furthermore, TM5 and TM7 peptide analogs had no or little effects on CXCR4 function and crystallographic structures revealed that CXCR4 exists as a homodimer in which TM5 and TM6 form the main interface between the receptor protomers, which make TM5/6 of CXCR4 as interaction sites with other GPCRs unlikely [31,32]. Therefore, we chose TM2 and TM4 peptide analogs for the present study as both peptides are known to inhibit CXCR4 [31,33]. This provides the advantage that possible differences in their effects on receptor heteromerization cannot be attributed to differences in their effects on CXCR4 function. Figure 1A shows typical proximity ligation assay (PLA) images for the detection of CXCR4:α_1A_-AR and CXCR4:ACKR3 interactions in human VSMC (hVSMC) incubated with or without the TM peptide analogs and Figure 1B,C show the quantification of the PLA signals from three independent experiments. As observed previously [30], we detected that a TM2 peptide analog reduced the PLA signals corresponding to CXCR4:α_1A_-AR complexes, but did not affect PLA signals for CXCR4:ACKR3 interactions (Figure 1B). In contrast, incubation of hVSMC with a TM4 peptide analog did not affect PLA signals for CXCR4:α_1A_-AR complexes, whereas PLA signals for CXCR4:ACKR3 complexes were significantly reduced (Figure 1C).

To address the possibility that the observed changes in PLA signals are caused by TM peptide analog-induced receptor internalization, we then studied receptor expression levels on hVSMC by FACS analyses. As shown in Figure 1D, incubation of hVSMC with the TM peptide analogs did not affect cell surface expression levels of CXCR4, ACKR3 or α_1A_-AR, when compared with untreated hVSMC. To confirm that the antibodies that we used for PLA and FACS analyses are suitable to detect changes in receptor expression levels [34,35], we then incubated hVSMC with non-targeting siRNA or siRNA specific for each receptor and measured receptor expression levels by FACS analyses. As shown in Figure 1E, all antibodies reported with a significant reduction of the fluorescence signals after incubation of hVSMC with the corresponding receptor-specific siRNA (% reduction in fluorescence signal *vs.* non-targeting siRNA: CXCR4 siRNA—73%, ACKR3 siRNA—72.5%, α_1A_-AR siRNA—70%).

**Figure 2:**
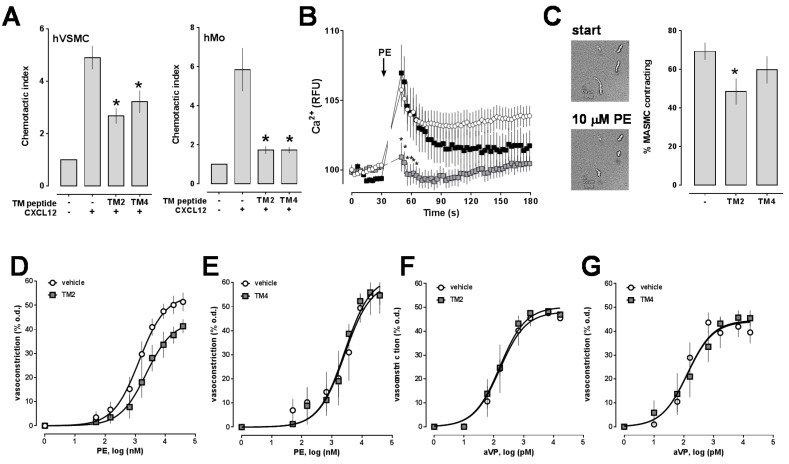
(**A**) Effects of the TM peptides on CXCL12 (100 nM, +; control: vehicle, −)-induced chemotaxis of hVSMC (*n* = 5; **left**) and freshly isolated human monocytes (hMo, *n* = 4; **right**). Cells were incubated in the absence (−, control) or presence of the TM peptides (100 μM). *: *p* < 0.05 *vs.* control; (**B**) Effects of the TM peptides on Ca^2+^ fluxes in A7r5 cells. Cells were pre-incubated with vehicle or 100 μM of the TM peptides for 15 min at room temperature. Arrows: Time point of phenylephrine administration (PE); Open circles: control (vehicle); Grey squares: Cells pretreated with the TM2 peptide; Black squares: Cells pretreated with the TM4 peptide. *n* = 3–4 independent experiments. *: *p* < 0.05 *vs.* vehicle and TM4; (**C**) **Left**: Typical images of MASMC before (start) and after phenylephrine (10 μM, PE) administration. Scale bar: 20 μm; **Right**: Quantification of the percentage of MASMC contracting upon stimulation with phenylephrine. Cells were pre-incubated with vehicle (−; control) or 100 μM of the TM peptides for 15 min at 37 °C. *: *p* < 0.05 *vs.* control; (**D**–**G**) Vasoconstriction was measured as change of the outer diameter (o.d.) in pressure myography experiments. Dose responses to phenylephrine (PE) in the presence (grey squares) and absence (vehicle, open circles) of the TM2 (*n* = 8, (**D**)) or TM4 (*n* = 4, (**E**)) peptide analogs (10 µM). Dose responses to arginine vasopressin (aVP) in the presence (grey squares) and absence (vehicle, open circles) of the TM2 (*n* = 4, (**F**)) or TM4 (*n* = 5, (**G**)) peptide analogs (10 µM).

Next, we studied how interference with receptor heteromerization by the TM-derived peptides influences receptor function. To assess the effects of the TM peptide analogs on CXCR4-mediated effects, we first measured CXCL12-induced chemotaxis as a functional read-out. As shown in Figure 2A, both TM peptide analogs inhibited migration of human VSMC and human monocytes towards CXCL12.

To assess the effects on α_1_-AR function, we then tested whether the TM peptide analogs affect signaling and function of VSMC upon exposure to the selective α_1_-AR agonist phenylephrine. The TM2 peptide analog significantly inhibited phenylephrine-induced Ca^2+^ mobilization, whereas the TM4 peptide analog did not (Figure 2B). Similarly, the TM2 peptide analog reduced the percentage of freshly isolated rat mesenteric artery smooth muscle cells contracting in response to phenylephrine, whereas the TM4 peptide analog had no effects, as compared to cells exposed to phenylephrine in the absence of TM peptide analogs (% of VSMC contracting in response to phenylephrine: control: 70% ± 4%; TM2: 48% ± 7% (*p* < 0.05 *vs.* control); TM4: 60% ± 7%; Figure 2C).

To confirm physiological relevance of these observations in VSMC, we then studied the effects of the TM peptide analogs on phenylephrine-induced constriction of isolated rat mesenteric arteries in pressure myography experiments. When tested in parallel experiments with arteries from the same animals, the EC_50_ for phenylephrine to induce vasoconstriction was 1.3 ± 0.04 μM and maximal constriction in response to phenylephrine was 54% ± 3% of the outer diameter (o.d.) in the absence of TM peptide analogs. In the presence of the TM2 peptide analog, the EC_50_ for phenylephrine was 2.3 ± 0.1 μM and maximal constriction 43% ± 3% of the o.d. (*p* < 0.001 *vs.* control, Figure 2D). In contrast, the TM4 peptide analog did not affect EC_50_ for phenylephrine or maximal constriction when compared with untreated arteries in parallel experiments (Figure 2E). None of the peptides affected arginine vasopressin-induced vasoconstriction in pressure myography experiments (Figure 2F,G).

### 2.2. Effects of CXCR4 Knockdown in Vascular Smooth Muscle

**Figure 3:**
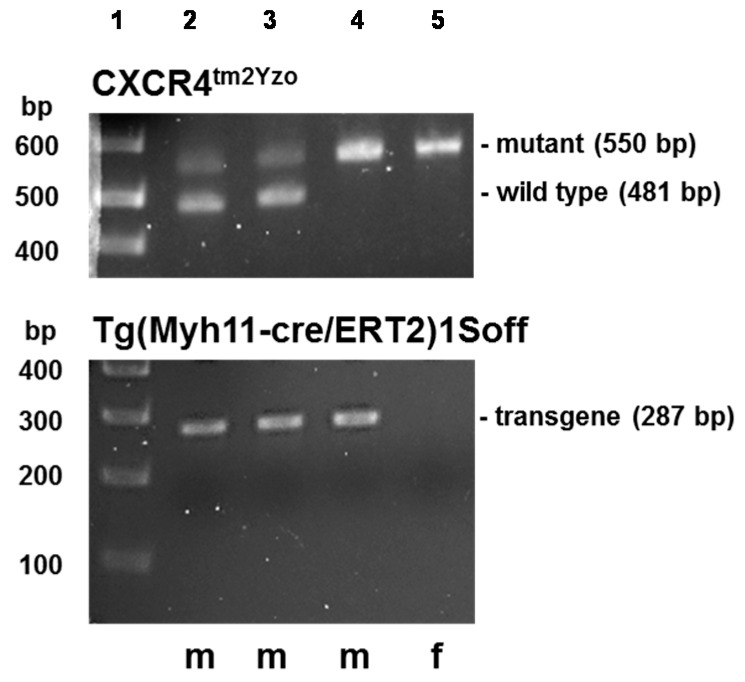
Genotyping of transgenic mice. Lane **1**: molecular weight markers; Lanes **2** and **3**: males, heterozygous for the CXCR4 loxP allele carrying the CreER^T2^ transgene on the Y chromosome; Lane **4**: male, homozygous for the CXCR4 loxP allele carrying the CreER^T2^ transgene in the Y chromosome = experimental animals; Lane **5**: female, homozygous for the CXCR4 loxP allele.

To provide proof-of-concept that CXCR4:α_1A/B_-AR heteromeric complexes are important for α_1_-AR function, we then sought to deplete CXCR4:α_1A/B_-AR heteromers in vascular smooth muscle utilizing a Cre-loxP mouse model that permits tissue specific and inducible knockdown of CXCR4 in smooth muscle. Mice carrying the CreER^T2^ transgene and homozygous for the CXCR4 loxP allele (Figure 3) developed without noticeable abnormalities. Similarly, after Cre induction with tamoxifen, there were no noticeable changes in behavior or food/water intake. Histology of tissues harvested 1–3 weeks after the last tamoxifen or vehicle injection did not reveal obvious differences between vehicle and tamoxifen injected animals (not shown).

**Figure 4:**
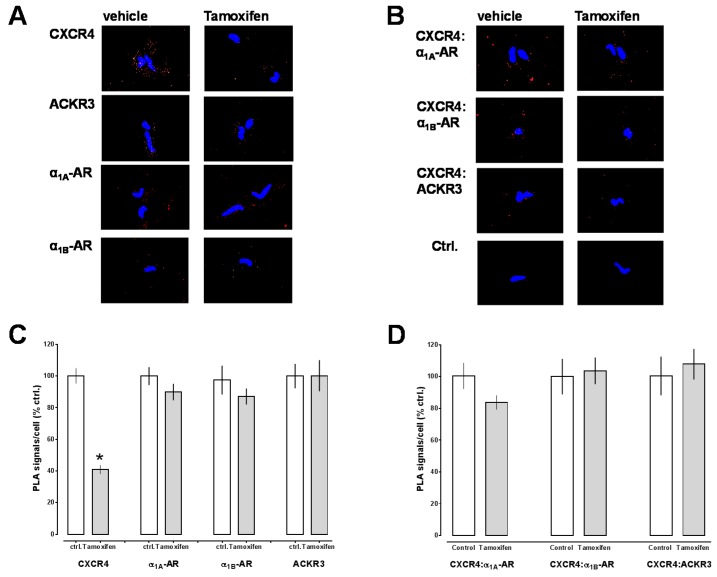
(**A**,**B**) Individual receptors (**A**) and interactions between CXCR4 and α_1A/B_-AR and between CXCR4 and ACKR3 (**B**) were visualized by PLA in VSMC from transgenic mice after treatment with vehicle (left) or tamoxifen (right). Images show merged PLA/DAPI signals. Ctrl.: Antibody control; the primary antibody was omitted; (**C**,**D**) Quantification of the number of PLA signals per cell for individual receptors (**C**) and receptor–receptor interactions (**D**) in VSMC from transgenic mice after treatment with vehicle (ctrl., open bars, *n* = 7) or tamoxifen (grey bars, *n* = 8). Data are expressed as % of vehicle treated animals. *: *p* < 0.05 *vs.* ctrl. (vehicle).

To document CXCR4 knockdown following tamoxifen injection, we then isolated aortic smooth muscle cells and analyzed receptor expression by PLA. Figure 4A,B shows typical PLA images for the detection of individual receptors (Figure 4A) and receptor interactions (Figure 4B) and Figure 4C,D the quantification of the PLA signals from 7–8 individual animals after tamoxifen or vehicle injection. As compared with vehicle injected animals, PLA signals for CXCR4 were reduced by 60% ± 3% after tamoxifen injection (*p* < 0.0001 *vs.* vehicle, Figure 4C). PLA signals for α_1A/B_-AR and ACKR3 in VSMC were not altered after Cre induction, as compared with vehicle injected animals (Figure 4C). When PLA was performed to detect receptor interactions (Figure 4D), we observed that signals for CXCR4:α_1A/B_-AR and CXCR4:ACKR3 interactions were not altered after tamoxifen injection, when compared with animals after vehicle injection.

**Figure 5:**
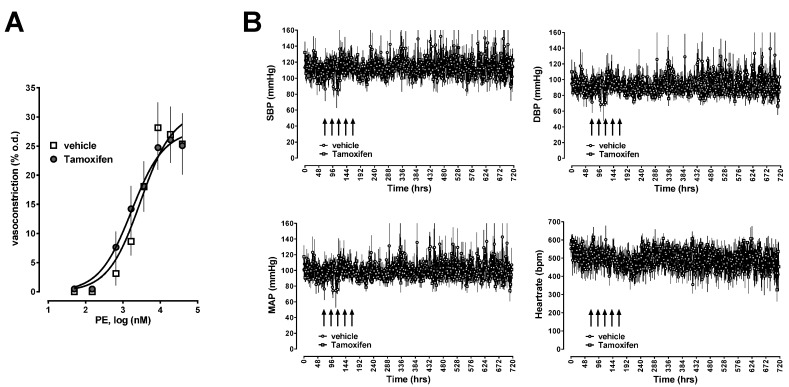
(**A**) Vasoconstriction was measured as change of the outer diameter (o.d.) in pressure myography experiments. Open squares: Mesenteric arteries from transgenic animals after vehicle injection, *n* = 12; Grey circles: Mesenteric arteries from transgenic animals after tamoxifen injection, *n* = 12; (**B**) Systolic (SBP), diastolic (DBP) and mean arterial blood pressures (MAP) in mmHg and heart rate (bpm, beats per minute) in transgenic animals after vehicle (open circles, *n* = 5) or tamoxifen (grey squares, *n* = 4) injection. Arrows indicate time points of tamoxifen/vehicle administration.

Furthermore, there were no differences in the reactivity of isolated mesenteric arteries to phenylephrine in pressure myography experiments (Figure 5A) and in long-term blood pressures or heart rates between animals injected with tamoxifen or vehicle (Figure 5B).

### 2.3. Structural Modeling and Molecular Dynamics Simulation of a Hetero-Hexameric Receptor Cluster

As our observations from experiments with TM peptide analogs point towards TM2 and TM4 of CXCR4 as contact sites in heteromeric receptor complexes, we then studied the structural configuration of the receptor heteromers *in silico*. We modeled a hetero-hexameric receptor cluster consisting of the CXCR4 homodimer, ACKR3 and α_1A_-AR. Because crystallographic structures of human ACKR3 and α_1A_-AR are currently not available, we modeled human ACKR3 and human α_1A_-AR structures and refined them by molecular dynamics simulation. We used the primary sequences of the receptors for secondary structure prediction followed by multiple sequence and structure alignments with other G protein-coupled receptors whose experimental structures are available via the Phyre2 server. The structural models of ACKR3 and α_1A_-AR were built based on human CXCR4 and β_2_-AR, respectively. The coverage of aligned sequences was 83% and 68% for ACKR3 and α_1A_-AR, respectively. The matched sequences were evenly distributed through the entire sequence. We visually inspected the models and confirmed that the transmembrane regions of the receptors of interest did not significantly diverge from template structures.

**Figure 6:**
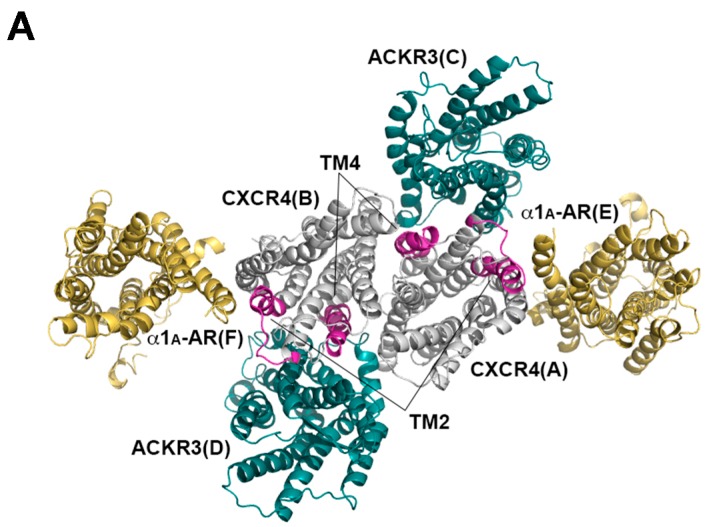

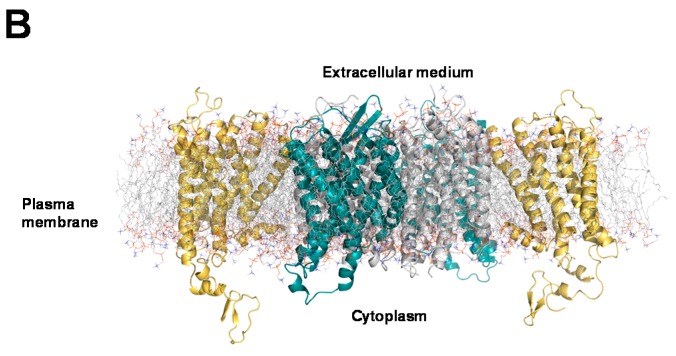
Structural modeling (**A**) and molecular dynamics simulations (**B**) of a hetero-hexameric receptor cluster consisting of the CXCR4, α_1A_-AR and ACKR3. Transmembrane (TM) helices of CXCR4 (gray), α_1A_-AR (yellow), and ACKR3 (green) are colored differently. TM helices of CXCR4 that participate in heteromerization interfaces with α_1A_-AR and ACKR3 are colored in purple.

For molecular dynamics simulations, we used the human CXCR4 homodimer (PDB ID: 3ODU) as the core of a hetero-hexameric receptor cluster. We assumed TM2 of CXCR4 as a likely contact site for α_1A_-AR and arranged the α_1A_-AR model next to TM2 of CXCR4. In an effort to guide this interaction based on an experimentally determined crystal structure, we utilized the turkey β_1_-AR dimer (PDB ID: 4GPO), whose dimerization interface is on the opposite side compared to the CXCR4 dimer, as a reference structure. We aligned the β_1_-AR subunit A (not shown) with the CXCR4 subunit A (Figure 6A) so the β_1_-AR subunit B could be used for the arrangement of the α_1A_-AR model. Next, based on the observed effects of the TM4 peptide, we arranged the ACKR3 model next to TM4 of CXCR4. In a similar manner as for α_1A_-AR, we aligned subunit A of the human histamine H1 receptor dimer (PDB ID: 3RZE) (not shown), whose TM4 is in its dimerization interface, with the CXCR4 subunit A so the histamine H1 receptor subunit B could be used for the arrangement of the ACKR3 model (Figure 6A). Likewise, the second set of α_1A_-AR and ACKR3 subunits were arranged next to the CXCR4 subunit B. Prior to molecular dynamics simulation, missing side chains were built by the Prime software and the hydrogen interaction was corrected. A predefined lipid bilayer, TIP3P water, and NaCl were included (Figure 6B).

**Figure 7:**
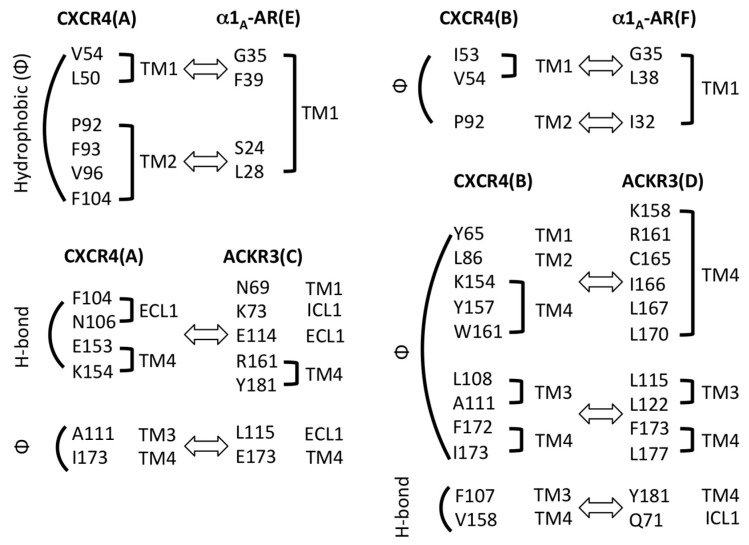
Contacts between CXCR4 and modeled receptors based on Figure 6. Receptor pairs are indicated in bold font. Hydrophobic and hydrogen (H)-bond interactions are grouped and indicated on the left side of each pair. Secondary structural location of each residue is indicated on the right side. Inter-receptor interactions are indicated with a block arrow.

Then, we energy minimized the initial model and performed molecular dynamics simulation by using the Desmond software from Schrödinger. We analyzed the quality of the resulting model using the built-in module in Maestro and did not find any steric clash between receptors. Non-covalent bonding interactions were analyzed and summarized in Figure 7. TM2 and TM4 of CXCR4 revealed major interactions with α_1A_-AR and ACKR3, respectively. The final structural coordinates of the receptor cluster (File S1; PyMol Session File) and a movie of the molecular dynamics simulation (File S2) are available as Supplemental Materials.

## 3. Discussion

In the present study, we further evaluated the physiological roles and regulation of CXCR4 heteromers in VSMC utilizing pharmacological and genetic approaches. There are several new findings from the present study. First, TM-derived peptide analogs of CXCR4 permit selective targeting of CXCR4 heteromers, suggesting TM2 and TM4 of CXCR4 as possible contact sites in heteromeric receptor complexes with α_1A_-AR and ACKR3, respectively; Second, a TM2 peptide analog of CXCR4, which interferes with CXCR4:α_1A_-AR heteromerization in isolated VSMC, inhibits α_1_-AR mediated constriction of intact isolated mesenteric arteries; Third, despite significant reduction of CXCR4 in VSMC in a Cre-loxP mouse model, signals corresponding to close proximity between CXCR4 and α_1A/B_-AR or ACKR3 in VSMC remained unaffected.

We have previously shown that heteromeric CXCR4:α_1A/B_-AR and CXCR4:ACKR3 complexes can be detected at a single molecule resolution by PLA [30,36]. As co-immunoprecipitation experiments in a HeLa expression system and in native VSMC suggested that the observed proximity between the receptors corresponds to direct receptor–receptor interactions [30], we utilized PLA to visualize and quantify interactions between CXCR4 and α_1A/B_-AR or ACKR3 in the present study.

Disruption of transmembrane domains of GPCRs with TM-derived peptides can inhibit receptor function and receptor dimerization through interference with the target membrane protein [37,38]. Our observations that the TM peptide analogs of CXCR4 inhibited CXCR4 mediated chemotaxis are consistent with previously described effects of the peptides on CXCR4 mediated Ca^2+^ fluxes in U87 cells stably expressing CXCR4 and on CXCR4 mediated cAMP levels in CXCR4 transfected HEK293T cells [36,37]. Furthermore, these data confirm our previous findings that a PEGylated TM2 peptide analog of CXCR4 reduced CXCR4:α_1A_-AR interactions without affecting CXCR4:ACKR3 heteromerization or expression levels of individual receptors [30].

The findings from the present study that the TM4 peptide analog interfered with CXCR4:ACKR3 heteromerization but did not affect CXCR4:α_1A_-AR heteromers or α_1_-AR-mediated effects have several implications. While the differential effects of the TM2 and TM4 peptide analogs on CXCR4 heteromerization serve as a rigorous control for each other, our findings also provide initial proof-of-concept that selective pharmacological targeting of a specific CXCR4 heteromer is possible. Furthermore, these observations suggest that the inhibitory effects of the TM2 peptide analog on α_1_-AR function are specific and can be attributed to selective interference with the formation of CXCR4:α_1A/B_-AR heteromeric complexes [30].

Our findings on the effects of the TM2 peptide analog from pressure myography experiments are in agreement with the effects of this peptide on VSMC and now provide evidence for inhibitory effects on intrinsic α_1_-AR function in intact arteries. As the TM2 peptide analog did not affect arginine vasopressin-induced vasoconstriction in pressure myography experiments, these data underline specificity for α_1_-AR and are consistent with the lack of effect of the PEGylated form of this peptide on arginine vasopressin-induced Ca^2+^-fluxes in VSMC that we described previously [30].

The effects of the TM-derived peptide analogs of CXCR4 on CXCR4 heteromerization point towards TM2 and TM4 as possible CXCR4 contact sites in heteromeric receptor complexes. Heteromerization of CXCR4 with ACKR3 via TM4 or with α_1A_-AR via TM2 may not interfere with the constitutive homodimeric CXCR4 complex, and thus could provide a conceivable structure-based explanation for the formation of higher order hetero-oligomeric CXCR4 complexes [32]. As information on the structural configuration of higher-order receptor heteromers is currently not available, we developed *in silico* a structure-based hetero-hexameric receptor model, in which TM2 and TM4 of CXCR4 participate in heteromerization with α_1A_-AR and ACKR3, respectively. This model suggests that such a receptor configuration is possible and now provides testable hypotheses that await further validation. Crystallographic structures of heteromeric receptor complexes, however, will ultimately be required to confirm the exact contact sites in CXCR4.

While effects of TM2- and TM4-derived peptide analogs of CXCR4 on other native receptor heteromers are unknown, TM2 and TM4 peptide analogs have previously been reported not to interfere with the CXCR4 homodimer or the CXCR4:CCR2 heterodimer bioluminescence resonance energy transfer (BRET) signals in a HEK293T expression system [33]. The TM peptides, however, reduced CXCL12-promoted BRET changes of the homo- and heteromeric receptors, suggesting that the peptides bound to CXCR4. Therefore, it could be speculated that CCR2 forms another interface with CXCR4, which is distinct from the interfaces for α_1A_-AR and ACKR3 [13,33]. Whether TM2 and TM4 of CXCR4 could also be involved in heteromeric complex formation between CXCR4 and other GPCRs, such as CCR5, CXCR3, ChemR23, β_2_-AR or α_1B_-AR [16,27,30,39,40], remains to be evaluated.

To gain initial information on the physiological role of CXCR4:α_1A/B_-AR heteromers on vascular function, we then utilized a Cre-loxP mouse model to delete CXCR4 in smooth muscle. CreER^T2^ positive mice, in which Cre-mediated recombination occurs exclusively in smooth muscle cells, have been used previously to study various aspects of vascular smooth muscle function [41]. As expected, CXCR4 expression in VSMC was significantly reduced after tamoxifen injection in CreER^T2^ positive mice homozygous for the CXCR4 loxP allele, as compared with mice after injection of vehicle. Quantification of the degree of knockdown revealed 60% reduction of CXCR4 expression on VSMC. This indicates incomplete excision of the loxP-flanked gene after Cre induction and is in agreement with incomplete deletion of loxP-flanked genes that have been observed in other transgenic mouse models utilizing smooth muscle myosin heavy chain CreER^T2^ [42,43,44,45].

The finding that CXCR4:α_1A/B_-AR and CXCR4:ACKR3 heteromeric complexes in VSMC were not reduced, despite significant reduction of PLA signals for CXCR4, was unexpected. We previously showed that *CXCR4* gene silencing in cultured human VSMC resulted in reductions of PLA signals for CXCR4 heteromers, in proportion to the degree of CXCR4 knockdown [30]. Our observations from the present study suggest that the relative abundance of CXCR4 that is expressed as a protomer or homodimer alone, compared with CXCR4 expressed within a heteromeric receptor complex, may not be a direct function of total CXCR4 expression levels. While the underlying molecular mechanisms remain to be determined, our findings could be interpreted as a preferential formation of CXCR4 heteromers, e.g., due to the interaction affinity between the receptors. According to this interpretation, free CXCR4 protomer/homodimers would occur only when CXCR4 is present in excess of the interacting receptor partners. Thus, the effects of CXCR4 knockdown on CXCR4 heteromerization would depend on the ratio between the expression levels of CXCR4 and the interacting receptor partners; PLA signals for CXCR4 heteromers would remain constant as long as the molecular ratio is high. Our previous finding that *CXCR4* gene silencing reduced PLA signals for CXCR4 heteromers in cultured human VSMC may not be contradictory because receptor expression levels in freshly isolated mouse VSMC that we used in the present study, and in cultured human VSMC that we utilized previously, could be substantially different [30].

Alternatively, it could be speculated that regulatory mechanisms occur in our genetic model, which promote CXCR4 heteromerization, and that such regulation is absent in cell cultures. Nevertheless, our finding that phenylephrine-reactivity of isolated arteries in pressure myography experiments and blood pressures of conscious mice were not affected after reduction of CXCR4 in VSMC is consistent with the maintenance of CXCR4: α_1A/B_-AR heteromer expression levels in VSMC in this transgenic mouse model.

Although our observations provide initial insights into the regulation of receptor heteromerization, our experimental approach was unable to evaluate the physiological function of CXCR4:α_1A/B_-AR heteromers in VSMC as those were maintained after CXCR4 knockdown. This documents an unexpected outcome of a genetic animal model despite a straight forward experimental approach that was based on findings from cell culture experiments. Thus, genetic models that permit complete CXCR4 knockdown to abolish CXCR4:α_1A/B_-AR heteromers or in which mutated CXCR4 that is unable to interact with α_1A/B_-AR is expressed in VSMC will be required to answer this question in the future.

In conclusion, our findings point towards TM2 and TM4 of CXCR4 as possible contact sites for heteromerization with α_1A/B_-AR and ACKR3, respectively, and suggest that TM-derived peptides permit selective targeting of CXCR4 heteromers. We anticipate that further development and refinement of TM-derived peptide analogs or small molecules that target specific receptor–receptor interaction sites can serve as useful tools to clarify the roles and mechanisms of GPCR heteromerization and may lead to the development of new compounds with therapeutic applicability. Our findings further support the notion that CXCR4:α_1A/B_-AR heteromers are important for intrinsic α_1_-AR function in intact arteries and provide initial and unexpected insights into the regulation of CXCR4 heteromerization in VSMC.

## 4. Materials and Methods

### 4.1. Ethics Statement

All procedures involving healthy blood donors were conducted in accordance with the Declaration of Helsinki-Ethical Principles for Medical Research Involving Human Subjects and the National Institutes of Health human subjects policies and guidance and were approved by the Loyola University Chicago Health Sciences Division Institutional Review Board for the Protection of Human Subjects. Informed consent was obtained from all blood donors. All procedures involving animals were conducted in accordance with the Guide for the Care and Use of Laboratory Animals, 8th Edition and were approved by the Institutional Animal Care and Use Committee of Loyola University Chicago (#205102, 10 July 2013). 

### 4.2. Proteins and Reagents

Phenylephrine was purchased from Sigma-Aldrich (St. Louis, MO, USA) and CXCL12 from Protein Foundry, Milwaukee, WI, USA. siRNA reagents were purchased from GE Dharmacon (Lafayette, CO, USA). The peptide analogs of transmembrane helix 2 (TM2) (LLFVITLPFWAVDAVANWYFGNDD-NH_2_) and TM4 (VYVGVWIPALLLTIPDFIFANDD-OH) of CXCR4 were generated using the Fmoc protected amino acids in a solid-phase synthesis on a 433A Applied Biosciences Peptide Synthesizer and Liberty Blue Microwave peptide synthesizer (CEM Corporation, Charlotte, NC, USA) using Fmoc chemistry. The peptides were cleaved from the resin and deprotected with a mixture of 90.0% (*v*/*v*) trifluoroacetic acid (TFA) with 2.5% water, 2.5% triisopropyl-silane, 2.5% 2,2′-(ethylenedioxy)diethanethiol and 5% thioanisol. Peptides were purified on a preparative (25 mm × 250 mm) Atlantis C3 reverse phase column (Agilent Technologies, Santa Clara, CA, USA) in a 90 min gradient of 0.1% (*v*/*v*) trifluoroacetic acid in water and 0.1% trifluoroacetic acid in acetonitrile, with a 10 mL/min flow rate. The fractions containing peptides were analyzed on Agilent 6100 LC/MS spectrometer (Agilent Technologies, Santa Clara, CA, USA) with the use of a Zorbax 300SB-C3 PoroShell column and a gradient of 5% acetic acid in water and acetonitrile. Fractions that were more than 95% pure were combined and freeze dried.

### 4.3. Cells and Cell Lines

Human aortic vascular smooth muscle cells (hVSMC) and the A7r5 rat aortic smooth muscle cell line were obtained from American Type Culture Collection (ATCC, Manassas, VA, USA) and cultured as described [30]. Mesenteric artery smooth muscle cells (MASMC) were isolated from male Sprague-Dawley rats and aortic smooth muscle cells were isolated from transgenic mice as described elsewhere [46,47,48]. In brief, small pieces (3–5 mm) of artery were cleaned from connective tissue and placed in a glass vial containing 0.5 mL of 140 mM NaCl, 5.36 mM KCl, 0.34 mM Na_2_HPO_4_, 0.44 mM KH_2_PO_4_, 1.2 mM MgCl_2_, 0.05 mM CaCl_2_, 10 mM HEPES and 10 mM d-Glucose at pH 7.2, 298 mOsm and incubated for 30 min at 37 °C. Arteries were then digested in the same buffer containing 2 mg/mL collagenase Type XI, 2 mg/mL BSA fraction V and 0.16 mg/mL elastase type IV for 35 min at 37 °C. After digestion, arteries were washed and triturated with a Pasteur pipette. Freshly isolated MASMCs were kept on ice until use in contraction assays.

Human monocytes were isolated from whole blood from healthy volunteers. In brief, whole blood was drawn and peripheral blood mononuclear cells were isolated by standard density gradient centrifugation. Monocytes were then positively selected using the EasySepTM Human CD14 positive selection kit (Stemcell Technologies, Vancouver, BC, Canada) and suspended in RPMI supplemented with 10% fetal bovine serum, 100 U/mL penicillin and 100 µg/mL streptomycin.

### 4.4. Transgenic Mice

Female B6.129P2-Cxcr4^tm2Yzo^/J mice and male B6.FVB-Tg(Myh11-cre/ERT2)1Soff/J were purchased form Jackson Laboratory (Bar Harbor, ME, USA) and mated. B6.129P2-Cxcr4^tm2Yzo^/J mice possess loxP sites on either side of exon 2 of the *CXCR4* gene [49]. In B6.FVB-Tg(Myh11-cre/ERT2)1Soff/J mice, CreER^T2^ expression, a fusion protein of the Cre recombinase with the modified estrogen receptor binding domain, is under control of the mouse smooth muscle myosin, heavy polypeptide 11, smooth muscle (Myh11) promoter/enhancer regions on the bacterial artificial chromosome (BAC) transgene, which is inserted on the Y chromosome [41]. Male offspring heterozygous for the loxP allele were then mated back to female B6.129P2-Cxcr4^tm2Yzo^/J mice. Male progeny carrying the CreER^T2^ transgene and homozygous for the loxP allele were then mated to female B6.129P2-Cxcr4tm2Yzo/J mice and male progeny selected as experimental animals. For Cre-induction, mice ≥12 weeks-old were injected intraperitoneally with 1 mg/kg body weight tamoxifen or vehicle (corn oil) for five consecutive days. Tissues were harvested not earlier than 7 days after the final tamoxifen injection.

### 4.5. Genotyping

Genotyping of transgenic mice with DNA extracted from tail-snips was performed using standard PCR according to the Jackson Laboratory protocols Cxcr4^tm2Yzo^ (primers: 5′-CCA CCC AGG ACA GTG TGA CTC TAA-3′ and 5′-GAT GGG ATT TCT GTA TGA GGA TTA GC-3′; mutant gene: 550 bp; wild type: 481 bp) and Tg(Myh11-cre/ERT2)1Soff (primers: 5′-TGA CCC CAT CTC TTC ACT CC-3′ and 5′-AGT CCC TCA CAT CCT CAG GTT-3′; transgene: 287 bp).

### 4.6. Blood Pressure and Heart Rate Measurements via Telemetry

Direct blood pressure measurements in conscious mice were performed utilizing implantable radio telemetry devices, as described [50]. In brief, mice were anesthetized with isoflurane, the catheter of a Data Sciences International (DSI, St. Paul, MN, USA) PhysioTel PA-C10 pressure transmitter was inserted into the left common carotid artery and advanced in the aortic arch. The telemetry device was then implanted subcutaneously along the flank of the animal. Post-operatively, animals received three doses of ampicillin (25 mg/kg intramuscularly) in 8 h intervals and were kept on a rodent warming pad for 24 h. Seven days post telemetry device implantation, telemetry transmitters were activated and blood pressures and heart rates were averaged over 10 s in 2 h intervals via the DSI telemetry receiver and Dataquest A.R.T. ™ Gold Acquisition Version 4.30 software (DSI, St. Paul, MN, USA).

### 4.7. Pressure Myography

Pressure myography was performed as described in detail previously with slight modifications [51]. Animals were anesthetized with 3.5% isoflurane. The mesentery was immediately removed and placed in 130 mM NaCl, 4.7 mM KCl, 1.18 mM KH_2_PO_4_, 1.17 mM MgSO_4_·7H_2_O, 14.9 mM NaHCO_3_, 5.5 mM d-Glucose, 0.026 mM EDTA, 1.16 mM CaCl_2_ aerated with 95% O_2_, 5% CO_2_ at 37 °C. The animal was than euthanized by cardiectomy and bilateral decompression of the lungs. Third or 4th order mesenteric arteries were dissected free from the mesentery, mounted onto two glass cannulae with United States Pharmacopeia (USP) scale 11-0 sutures and pressurized as required in a DMT 110P Pressure myograph system (DMT-USA, Ann Arbor, MI, USA). The intraluminal solution and the vessel bath solution were the same as described before. The vessel bath solution was continuously aerated with 95% O_2_, 5% CO_2_ throughout the experiment. The outer o.d. of the pressurized vessel was then continuously measured and recorded via digital video-edge detection. For each condition or drug treatment, the vessel was observed for 7 min and the mean o.d. during the final minute of the dosing period calculated as percent of the o.d. before drug treatment.

### 4.8. Receptor Gene Silencing by RNA Interference

CXCR4, ACKR3 and α_1A_-AR siRNA gene silencing were performed as described previously [30,52]. In brief, hVSMC cells were grown in 1 mL Accell siRNA delivery media per well (Thermo Scientific Dharmacon, Waltham, MA, USA) in 12 well plates (Nunc, Rochester, NY, USA). Commercially available Accell CXCR4, ACKR3 and α_1A_-AR siRNA were reconstituted with siRNA buffer to a stock concentration of 100 µM. Cells were then transfected with 10 nmol CXCR4, ACKR3 or α_1A_-AR siRNA and incubated for 72 h at 37 °C, 5% CO_2_. Accell non-targeting (NT) siRNA pool was used as negative control. After 72 h, cells were assayed for receptor cell surface expression.

### 4.9. Vascular Smooth Muscle Cell Contraction Assay

Contraction assays were performed as described previously [30]. In brief, freshly isolated MASMCs were dispensed onto a glass coverslip base of the recording chamber and allowed to adhere for approximately 15 min at room temperature. MASMCs were pretreated with vehicle or 100 μM of the TM peptide analogs for 15 min at room temperature. All experiments were performed with continuous perfusion of the bath solution containing 140 mM NaCl, 5.36 mM KCl, 1.2 mM MgCl_2_, 2 mM CaCl_2_, 10 mM HEPES, and 10 mM d-glucose, pH 7.3, 298 mOsm/L. Images were acquired using C-Imaging System (Compix Inc., Lake Oawego, OR, USA) with an Olympus 1X71 inverted epifluorescence microscope (10× objective, phase contrast) and Simple PCI software (Vers.5.3.1., Olympus, Center Valley, PA, USA) every 2 s for 1 min prior to the application of 10 µM phenylephrine and for additional 3 min after phenylephrine application. The total number of the cells and the number of the cells that contracted in response to phenylephrine were counted in each field. For each experiment, cells from a single animal were tested in triplicate or quadruplicate.

### 4.10. Proximity Ligation Assays (PLA)

The Duolink proximity ligation assay was purchased from Sigma-Aldrich and performed in a format suitable for the detection of single proteins and protein-protein interactions, as described in detail previously [30,53,54]. In brief, hVSMC and mouse VSMC were fixed on eight well tissue culture slides (Nunc). After blocking in 3% BSA/PBS, hVSMC were incubated with rabbit anti-ACKR3 (LS-B1815, LSBio, Seattle, WA, USA)/mouse anti-CXCR4 (M04, clone 2G9, Abnova, Walnut, CA, USA) (1:400) or mouse anti-CXCR4 (M04, clone 2G9, Abnova)/rabbit anti-α_1A_-AR (ab137123, Abcam, Cambridge, MA, USA) (1:400) antibodies at 37 °C for 2 h in a humidifying chamber to visualize receptor–receptor interaction. Mouse VSMC were incubated either with rabbit anti-CXCR4 (AbCam ab2074) (1:500), mouse anti-ACKR3 (R&D Systems MAB42273, Minneapolis, MN, USA) (1:500), mouse anti-α_1A_-AR (AbCam ab87990) (1:500) or goat anti-α_1B_-AR (Santa Cruz sc27136, Dallas, TX, USA) for detection of individual receptors or with the following antibody combinations (all 1:500) for the detection of receptor–receptor interactions: rabbit anti-CXCR4 (AbCam ab2074)/mouse anti-ACKR3 (R&D Systems MAB42273), rabbit anti-CXCR4 (AbCam ab2074)/mouse anti-α_1A_-AR (AbCam ab87990) and rabbit anti-CXCR4 (AbCam ab2074)/goat anti-α_1B_-AR (Santa Cruz sc27136). Slides were then incubated with secondary anti-species specific antibodies conjugated with plus and minus Duolink II PLA probes (1:5), as appropriate. As controls, slides were incubated with PBS instead of primary antibody or with rabbit anti-CXCR4 (AbCam ab2074) in combination with a single secondary probe. After washing, slides were then incubated with ligation-ligase solution (30 min, 37 °C) followed by incubation with amplification-polymerase solution (2 h, 37 °C). Slides were then mounted with a minimal volume of Duolink II Mounting medium with 4′,6-diamidino-2-phenylindole (DAPI) for 15–30 min and PLA signals (Duolink In Situ Detection Reagents Green (λ_excitation_/_emission_ 495/527 nm) or Red (λ_excitation_/_emission_ 598/634 nm)) were identified as fluorescent spots under a fluorescence microscope using 40× or 100× objectives. The number of PLA signals per cell were analyzed using the Duolink Image Tool software (Sigma-Aldrich). Images were imported in merged.tiff formats containing both signal and nuclei channels. Merged images were checked visually and verified for analytical quality. Comparisons and statistical analyses were performed only when PLA assays were performed on the same day in parallel experiments and fluorescence microscopy was performed with identical settings. For hVSMC, 10 vision fields were analyzed for each condition and experiment. For mouse VSMC, 6–15 vision fields were analyzed for each condition and animal (*n* = 7–8).

### 4.11. Fluorescence-Activated Cell Sorting (FACS) Analyses

FACS analyses were performed as described previously [30,53]. Cells were labeled with monoclonal mouse anti-CXCR4 (M04, clone 2G9, Abnova), polyclonal rabbit anti-α_1A_-AR (ab137123, Abcam) and polyclonal rabbit anti-ACKR3 (LS-B1815, LSBio) in combination either with anti-rabbit FITC conjugated goat IgG (ab6717, Abcam) or anti-mouse FITC conjugated goat IgG (ab6785). Rabbit IgG (GWB-3274CD, R&D Systems) in combination with FITC conjugated anti-rabbit goat IgG (ab6717, Abcam) was used as a negative control. The geometric fluorescence intensities of at least 3 × 10^4^ cells were recorded and analyzed using the FlowJo software (Tree Star, Ashland, OR, USA).

### 4.12. Ca^2+^ Assays

Intracellular Ca^2+^ in A7r5 cells was measured using the Fluo-4 NW Calcium Assay Kit (Molecular Probes, Eugene, OR, USA), as described [30,52,55,56].

### 4.13. Chemotaxis Assay

Cell migration of hVSMC and monocytes towards CXCL12 was assessed using the ChemoTx96-well cell migration system (30 µL well plate, 8 µm filter pore size; Neuroprobe, Gaithersburg, MD, USA). ChemoTX filters were pre-coated with 100 µg/mL Collagen IV for 48 h at 25 °C when hVSMC were tested. The bottom wells were loaded with 30 µL of test solutions (100 ng/mL CXCL12). Twenty-five microliters of cell suspension containing 10^5^ cells were loaded onto the ChemoTX filter over each well and incubated for 2 h at 37 °C, 5% CO_2_. After incubation, transmigrated cells were washed, fixed and stained with Accustain Wright-Giemsa stain (Sigma-Aldrich). Stained cells were counted under a light microscope (400×) and average cell numbers were determined by counting the cells in three random non-overlapping vision fields. The chemotactic index was calculated as the ratio of cells that transmigrated through the filter in the presence *versus* absence (=PBS/control) of the test solutions.

### 4.14. Light Microscopy

Tissue biopsies (lung, heart, liver, kidney, gut, urinary bladder and vasculature) were fixed in 10% formalin, embedded in paraffin, sectioned and stained with hematoxylin and eosin (H&E). Slides were examined under light microscopy by a pathologist who was blinded as to the origin of the tissues.

### 4.15. Sequence Alignments

Structure-based sequence alignments were performed with the web-based GPCRdb similarity search tool (Available online: http://gpcrdb.org/), where sequence similarity is defined as the percentage of similar amino acids and similar is defined as a BLOSUM62 score >0 [57,58].

### 4.16. Structural Modeling and Molecular Dynamics Simulation

Structural modeling of ACKR3 and α_1A_-AR were conducted via the Protein Homology/analogY Recognition Engine (Phyre2) server [59]. Experimental and modeled structures were aligned in PyMol (Schrödinger, New York, NY, USA) prior to molecular dynamics simulation. Molecular dynamics simulation was carried out using Desmond (Schrödinger) installed on the Discovery fast computing server at the Edward A. Doisy Department of Biochemistry and Molecular Biology at the Saint Louis University School of Medicine.

### 4.17. Data Analyses

Data are expressed as mean ± standard error of the mean (SEM) from *n* independent experiments that were performed on different days. Data were analyzed with non-linear regression analyses and with one-way analyses of variance (ANOVA) with Bonferroni’s multiple comparison *post-hoc* test or with two-way ANOVA with Sidak’s multiple comparisons test for multiple comparisons, as appropriate. Best-fit values were compared with the extra sum-of-squares *F* test. All analyses were calculated with the GraphPad-Prism 6 software (GraphPad Software, La Jolla, CA, USA). A two-tailed *p* < 0.05 was considered significant.

## Supplementary Materials

Supplementary materials can be found at http://www.mdpi.com/1422-0067/17/6/971/s1. File S1: Final structural coordinates of the hetero-hexameric receptor cluster (PyMol file S1.pse); File S2: Movie (sampling interval 4.8 ps, 250 frames) of the molecular dynamics simulation (S2.mpeg).

## Abbreviations

ACKR3	Atypical chemokine receptor 3
AR	Adrenergic receptor
aVP	arginine vasopressin
CCR	chemokine (C-C motif) receptor
Chem23R	G protein-coupled receptor 27, chemerin receptor
Cre	Cre recombinase
CreER^T2^	Cre recombinase fused to a triple mutant form of the human estrogen receptor
CXCR4	chemokine (C-X-C motif) receptor 4
DAPI	4′,6-diamidino-2-phenylindole
DBP	diastolic blood pressure
EC_50_	half-maximal effective concentration
FACS	Fluorescence-activated cell sorting
GPCR	G protein-coupled receptor
loxP	locus of crossover in P1
MAP	mean arterial blood pressure
o.d.	outer diameter
PE	Phenylephrine
PLA	proximity ligation assay
PCR	polymerase chain reaction
siRNA	small interfering ribonucleic acid
SBP	systolic blood pressure
TIP3P	water model
TM	transmembrane-domain
VSMC	vascular smooth muscle cell
